# A guide to chemokines and their receptors

**DOI:** 10.1111/febs.14466

**Published:** 2018-04-24

**Authors:** Catherine E. Hughes, Robert J. B. Nibbs

**Affiliations:** ^1^ Institute of Infection, Inflammation & Immunity College of Medical, Veterinary and Life Sciences University of Glasgow UK

**Keywords:** atypical chemokine receptor, cell migration, chemokine, chemokine receptor, glycosaminoglycan, immune surveillance, inflammation, leukocyte, oligomerization, protease

## Abstract

The chemokines (or chemotactic cytokines) are a large family of small, secreted proteins that signal through cell surface G protein‐coupled heptahelical chemokine receptors. They are best known for their ability to stimulate the migration of cells, most notably white blood cells (leukocytes). Consequently, chemokines play a central role in the development and homeostasis of the immune system, and are involved in all protective or destructive immune and inflammatory responses. Classically viewed as inducers of directed chemotactic migration, it is now clear that chemokines can stimulate a variety of other types of directed and undirected migratory behavior, such as haptotaxis, chemokinesis, and haptokinesis, in addition to inducing cell arrest or adhesion. However, chemokine receptors on leukocytes can do more than just direct migration, and these molecules can also be expressed on, and regulate the biology of, many nonleukocytic cell types. Chemokines are profoundly affected by post‐translational modification, by interaction with the extracellular matrix (ECM), and by binding to heptahelical ‘atypical’ chemokine receptors that regulate chemokine localization and abundance. This guide gives a broad overview of the chemokine and chemokine receptor families; summarizes the complex physical interactions that occur in the chemokine network; and, using specific examples, discusses general principles of chemokine function, focusing particularly on their ability to direct leukocyte migration.

**Abbreviations**
ACKR: atypical chemokine receptor
ADM: adrenomedullin
ADRA1A/B: α1A/B‐adrenoreceptors
BM: bone marrow
C‐18: cyclophilin‐18 of *T. gondii*
CB2: cannabinoid receptor 2
cCKR: conventional chemokine receptor
CCL: CC chemokine ligand
CCR: CC chemokine receptor
CNS: central nervous system
CX_3_CL: CX_3_C chemokine ligand
CX_3_CR: CX_3_C chemokine receptor
CXCL: CXC chemokine ligand
CXCR: CXC chemokine receptor
DBP: Duffy binding protein
DC: dendritic cell
DOR: delta‐opioid receptor
ECM: extracellular matrix
FPRL1: formyl peptide receptor‐like 1
GAG: glycosaminoglycan
GluR1: component of the AMPA‐type glutamate receptor
Glyco G: RSV G glycoprotein
gp120: envelope protein of HIV
GPCR: G protein‐coupled receptor
GPR75: G protein‐coupled receptor 75
HIV: human immunodeficiency virus
HlgAB: *Staphylococcus aureus* γ‐Hemolysin AB
HMGB1: high mobility group box 1 protein
HSC: hematopoietic stem cell
KOR: kappa‐opioid receptor
LEC: lymphatic endothelial cell
LTi: lymphoid tissue inducer
LukED: *Staphylococcus aureus* leukotoxin ED
MIF: macrophage migration inhibitory factor
MMP: matrix metalloproteinase
MOR: mu‐opioid receptor
PITPMN3: phosphatidylinositol transfer protein 3
PSMB: PC3‐secreted microprotein
RSV: respiratory syncytial virus
TCR: T‐cell receptor
Treg: regulatory T cell
TSG‐6: TNF‐stimulated gene 6
WHIM: warts, hypogammaglobulinemia, immunodeficiency, and myelokathexis
XCL: XC chemokine ligand
XCR: XC chemokine receptor
β2AR: β2‐adrenergic receptor

## Chemokines

**Figure 1:**
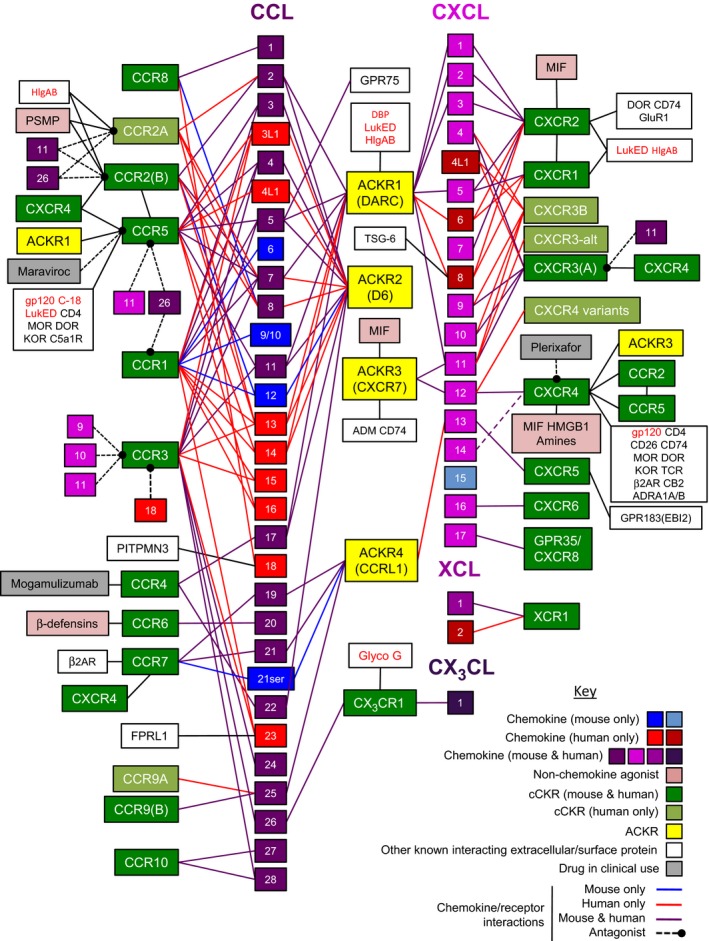
Mammalian chemokine receptors and their known interactions with chemokines and other key secreted, cell surface, and pathogen‐encoded molecules. Chemokines of the four subclasses (CCL, CXCL, CX_3_ CL, and XCL) are arranged numerically in columns and represented as numbered squares that are color‐coded according to whether they are in humans and mice, humans only, or mice only (see Key). The chemokine–chemokine ‘interactome’ 40 is not depicted. Chemokines are linked by lines to receptors that they are known to bind: yellow boxes are atypical chemokine receptors (ACKRs) (previous names shown in parentheses); green boxes are conventional chemokine receptors (cCKRs); and light green boxes show reported human cCKR variants generated by alternative splicing at the N terminus (CCR9, CXCR3, CXCR4) or C terminus (CCR2) 69, 70, 71, 72, 73, 74. The color of the linking line (see Key) indicates whether the interaction likely exists in humans only, mice only, or in humans and mice. Hashed black lines ending with a filled circle link chemokines with receptors they can antagonize 119, 120, 121, 122, 123, 124, 125, 126. CXCL14 is reported to be a positive allosteric modulator of CXCR4 344. Chemokine receptors reported to form heterodimers are linked with a black line 93, 94, 95, 96, 97, 98, 99, 100, 101, 118. Nonchemokine proteins in light pink boxes are able to activate the cCKR they are joined to by a black line 89, 90, 91, 92, 102, 347. White boxes contain microbial proteins (red text) and other host extracellular/surface proteins (black text; nonchemokine, nonchemokine receptor) that have been reported to interact, in the absence of chemokine, with the cCKR or ACKR to which they are attached by a black line [32, 92, 102, 103, 104, 105, 106, 107, 108, 109, 110, 111, 112, 113, 114, 115, 116, 117, 147, 148, 149, 150, 151, 152, 167, 233, 264, 345, 346, 347, 348]. Note that cCKRs and ACKRs other than those shown are known to be capable of binding HIV and/or gp120, but the role of these chemokine receptors during infection is uncertain. CCL18 receptor PITPMN3 and CCL5 receptor GPR75 are also shown 127, 348. FPRL1 interacts with a peptide released proteolytically from the N terminus of one form of CCL23 20. Gray boxes show drugs in clinical use: Maraviroc 143 and Plerixafor 237, antagonists of CCR5 and CXCR4, respectively; and Mogamulizumab, a humanized anti‐CCR4 antibody approved for treatment of relapsed or refractory CCR4+ adult T‐cell leukemia/lymphoma (CTCL) 349. Definitions: ADM, adrenomedullin; ADRA1A/B, α1A/B‐adrenoreceptors; C‐18, cyclophilin‐18 of *Toxoplasma gondii;* CB2, cannabinoid receptor 2; DBP, Duffy binding protein of malarial parasites *P. vivax* and *P knowlesi;* DOR, delta‐opioid receptor; GluR1, component of the AMPA‐type glutamate receptor; Glyco G, RSV G glycoprotein; gp120, the gp120 envelope protein of HIV; GPR75, G protein‐coupled receptor 75; HlgAB, *Staphylococcus aureus* γ‐Hemolysin AB; HMGB1, high mobility group box 1 protein; KOR, kappa‐opioid receptor; LukED, *S. aureus* leukotoxin ED; MIF, macrophage migration inhibitory factor; MOR, mu‐opioid receptor; PITPMN3, phosphatidylinositol transfer protein 3; PSMP, PC3‐secreted microprotein; TCR, T‐cell receptor; TSG‐6, TNF‐stimulated gene 6; β2AR, β2‐adrenergic receptor. Extended from previous reviews 64, 350.

Chemokines are defined by their primary amino acid sequence and the arrangement of specific structurally important cysteine residues within the mature protein. These form disulfide bonds that maintain the structure of the chemokine monomer, which consists of a central three stranded β‐sheet, an overlying C‐terminal α‐helix, and a short unstructured N terminus that plays a critical role in receptor activation 1. Variation in the precise configuration of the two cysteines closest to the N terminus allows chemokines to be split into four subfamilies: CC, CXC, CX_3_C, and XC. In CC chemokines, these cysteines are directly juxtaposed, while CXC chemokines have a single variable amino acid between them. The sole CX_3_C chemokine has three amino acids between these two cysteines, while XC chemokines, of which there are two forms in humans and one in mice, lack the first and the third cysteines of the motif. Large numbers of CC and CXC chemokine genes have been defined in many species (Fig. 1) 2: not all are found in all species, or sometimes all members of a species; nonallelic isoforms exist, such as *CCL3L1* and *CCL3* in humans 3, 4 and *Ccl21a*,* Ccl21b*, and *Ccl21c* in mice 5; and allelic and copy number variation creates considerable genetic diversity that influences susceptibility to, and severity of, a number of diseases 3, 6, 7.

Although chemokines were originally named according to specific functions, a systematic nomenclature was introduced in 2000 that includes a subfamily designation (i.e., CC, CXC, CX_3_C, or XC), followed by the letter L (denoting ‘ligand’), and then a number according to when the gene was first isolated 8, 9. Chemokines with the same name from different species are often functional orthologues 2, although this is not always the case: for example, human CCL8 binds to the human CCR2 receptor, while mouse CCL8 is a CCR8 ligand 10, and mouse CCL3 is functionally more like human CCL3L1 than human CCL3 11. All chemokines are produced with an N‐terminal signal peptide that is removed once it has directed the chemokine into the endoplasmic reticulum for secretion. Two chemokines, CX_3_CL1 and CXCL16, have an extended C terminus containing a mucin‐like stalk and a transmembrane domain 12, 13. This holds these chemokines on the cell surface but can be proteolytically cleaved to release the chemokine portion into the extracellular space 14, 15, 16, 17, 18. Other chemokines, such as CCL6, CCL9, and CCL23, have an extended N terminus that can be proteolytically removed to enhance receptor activation capabilities 19. An N‐terminal peptide cleaved off a CCL23 variant can activate formyl peptide receptor‐like 1 (FPRL1), a G protein‐coupled receptor (GPCR) not classified as a cCKR 20. Alternatively spliced transcripts can generate chemokine variants: for example, six forms of human CXCL12 have been described with different C termini 21 and distinct biological properties 22, 23.

## GAGs, oligomerization, and post‐translational modification

The postsecretion activity and distribution of chemokines depends on how readily they become immobilized on cell surfaces and ECM 24, 25. Glycosaminoglycans (GAGs) are particularly important in this regard. Their ability to bind chemokines influences chemokine/receptor interactions; chemokine half‐life in a tissue or tissue compartment; how, and where, a chemokine operates *in vivo*; and the type of cell movement or adhesion it stimulates 24, 25. They are essential for maintaining interstitial chemokine functions and gradients 26, 27, 28 and for the presentation of chemokines on endothelial surfaces, preventing them being washed away by the blood and so allowing them to drive leukocyte arrest and extravasation 29, 30, 31. Some chemokines, such as CCL21, are very ‘sticky’ and become rapidly immobilized, while others, such as CCL19, which activates the same receptor as CCL21, likely diffuse more readily through tissues 27. In addition, mammalian proteins, including TSG‐6, can interfere with chemokine/GAG interactions to alter chemokine distribution and function 32, 33. Targeted disruption of chemokine/GAG interactions might have therapeutic impact 34, as could GAG‐based chemokine‐capturing hydrogels 35.

While chemokines are active as monomers, they also form homodimers, heterodimers, and higher order aggregates that can contain one or more chemokine species, and this can be influenced by interactions with GAGs 36, 37, 38, 39, 40. The full chemokine ‘interactome’ reveals complex and extensive interactions: human CXCL4 and CCL5, for example, can each heterodimerize/oligomerize with over 20 other chemokines from CC, CXC, and XC subfamilies 40. The structures formed rely on two types of interfaces, referred to as CC‐ and CXC‐type, in which chemokine activity is typically enhanced and inhibited, respectively 40. Oligomerization clearly influences how individual or mixtures of chemokines combine to control leukocyte responses, and disrupting specific interactions may have therapeutic potential 1, 25, 39, 40, 41.

Chemokines are also profoundly affected by post‐translational modifications such as citrullination 42, 43, 44, nitration/nitrosylation 45, 46, 47, and cleavage by matrix metalloproteinases (MMPs), cathepsins, thrombin, plasmin, elastase, the dipeptidyl peptidase CD26, and other proteases 48, 49, 50. These changes can substantially modify chemokine activity. For example, nitration of tyrosine residues in CCL2 by reactive nitrogen species reduces the ability of this chemokine to attract monocytes through its receptor CCR2 45, while arginine residues in a number of chemokines can be converted into citrulline by the enzyme peptidylarginine deiminase: this reduces the chemotactic activity of CXCL8, CXCL10, and CXCL11, and prevents conversion of CXCL8 into a more active shortened form by interfering with thrombin‐ and plasmin‐mediated N‐terminal trimming 42, 44.

Proteases are key chemokine regulators. CD26‐mediated trimming of two amino acids off a chemokine's N terminus can, depending on the chemokine, change receptor specificity, substantially alter receptor affinity or convert agonists into antagonists 48. Many MMPs can also modify this part of certain chemokines, and the impact of these modifications depends on the identity of the chemokine being studied. Thus, MMP‐mediated N‐terminal trimming typically enhances the activity of CXCR2 ligands, and of CC chemokines with extended N‐termini (CCL6, CCL9, CCL23), while CXCL12 is inactivated by MMPs and CCR2 ligands are converted into receptor antagonists 50. Proteolytic cleavage of the C terminus of chemokines can dramatically alter ECM binding properties and diffusivity. For example, DC‐mediated cleavage of CCL21 removes the highly charged C terminus that anchors it to the ECM, thereby releasing a version of this key DC attractant that has much higher diffusivity than the full‐length protein 51. This is rather similar to the release of CX_3_CL1 and CXCL16 from cell surfaces that was mentioned above 14, 15, 16, 17, 18. MMPs and other proteases can also act on the C terminus of chemokines: for example, MMP processing of the C terminus of CCL16 enhances its GAG‐binding properties 52. Proteases are also thought to help create and modify interstitial chemokine gradients, and have the capacity to degrade chemokines, or the ECM to which they are bound, so are important in regulating chemokine half‐life and distribution 49, 53. Controlling protease regulation of chemokines could have therapeutic application: for example, inhibiting CD26‐mediated cleavage of CXCL10 enhances tumor immunotherapy in mouse models 54.

## Chemokine receptors

There are two families of heptahelical surface molecules that bind to chemokines: conventional chemokine receptors (cCKRs) and atypical chemokine receptors (ACKRs) (Fig. 1).

### Conventional chemokine receptors

Chemokine‐bound cCKRs typically transduce signals through pertussis toxin‐sensitive Gα_i_ G‐proteins and β‐arrestins, ultimately leading to cell migration, adhesion and/or a variety of other biological responses. Chemokines are thought to initially tether to their cognate cCKR via the extracellular loops and N terminus of the receptor: the negative charge on these cCKR domains can be increased by glycosylation, polysialylation, and/or the incorporation of sulphated tyrosine residues. Sulphated tyrosines in the N terminus aid HIV gp120 binding to CCR5 55 and enhance chemokine binding to CCR2 56, CCR3 57, CCR5 55, 58, CCR8 59, CXCR3 60, CXCR4 61, and CX_3_CR1 62. Polysialylation of CCR7 is essential for its activation by CCL21, appearing to release CCL21 from an auto‐inhibited conformation 63.

Once a chemokine is tethered to a cCKR, its unstructured N terminus enters the cCKR's heptahelical bundle to induce a conformational change that is translated into intracellular signals 64, 65. This classical two‐site model of chemokine/receptor interaction is probably oversimplistic, with recent studies suggesting that the two supposedly independent ligand‐binding sites can be physically and allosterically linked, and that additional interactions between chemokine and receptor are likely to be involved in ensuring full receptor activation 64. The signaling pathways downstream of chemokine receptors are complex, and a detailed description is beyond the scope of this review, but they include, among others, heterotrimeric G‐proteins, β‐arrestins, and JAK‐STAT pathways 66, 67.

There are currently 18 cCKRs named according to the predominant type of chemokine they bind (i.e., CC, CXC, CX_3_C, or XC), followed by the letter R (denoting ‘receptor’), and then a number reflecting the order of their discovery (green boxes, Fig. 1). There are 10 CCRs, 6 CXCRs, and a single CX_3_CR and XCR. GPR35 has recently been identified as a CXCL17 receptor and referred to as CXCR8 68, but may become known as CXCR7 now that the original CXCR7 has been renamed ACKR3 66. Transcripts encoding cCKRs can be subject to alternative splicing: variants of CCR2, CCR9, CXCR3, and CXCR4 have been reported with altered ligand‐binding or signaling properties 69, 70, 71, 72, 73, 74. Important detailed insights into cCKR structure, chemokine binding, and mechanisms of antagonism have come with the resolution of crystal structures of CCR2, CCR5 CCR9, CXCR4, and US28, a cytomegalovirus‐encoded chemokine receptor, and from other biophysical approaches 64, 65, 75, 76, 77, 78, 79, 80, 81, 82, 83, 84.

Receptor specificity is complex: many chemokines bind to multiple cCKRs, and some cCKRs have many ligands (Fig. 1). This is prominent among chemokines/cCKRs involved in inflammation, while those primarily involved in homeostatic cell migration have only one or two ligands that are faithful to a single cCKR. Chemokines vary with respect to their affinity for a particular cCKR, and biased signaling, or functional selectivity, is emerging as a key feature of cCKRs, such that the precise pathways activated by a cCKR depend on which ligand it binds, and the cellular context of that binding 85, 86, 87, 88. Currently, six cCKRs have been reported to show biased signaling 88. Moreover, some cCKRs can also be activated by nonchemokine ligands: β‐defensins can activate CCR6 89; the ‘alarmin’ high mobility group box 1 protein (HMGB1) is emerging as a key CXCR4 ligand 90, 91; and cells expressing CXCR2 or CXCR4 migrate in response to macrophage migration inhibitory factor (MIF) (light pink boxes, Fig. 1) 92.

Like other GPCRs, cCKRs exist as homodimers. They can also aggregate into higher order oligomers, and form functionally distinct heterodimers with ACKRs, other cCKRs, nonchemokine‐binding GPCRs (such as opioid receptors), and other membrane proteins (white boxes, Fig. 1) 87, 93, 94, 95, 96, 97, 98, 99, 100, 101, 102, 103, 104, 105, 106, 107, 108, 109, 110, 111, 112, 113, 114, 115, 116, 117, 118. Some chemokines can act as natural cCKR antagonists (Fig. 1) 119, 120, 121, 122, 123, 124, 125, 126. Phosphatidylinositol transfer protein (PITPMN3), a non‐GPCR with six transmembrane domains, is reported to be a functional receptor for CCL18 in the context of tumor cell invasion 127, although, more conventionally, CCL18 also binds to, and directs leukocyte migration through, the cCKR CCR8 128.

The striking receptor/ligand promiscuity common in the chemokine network most likely evolved to combat microbial subversion by building robustness into leukocyte responses during infection. Many viral genomes carry genes encoding chemokines, chemokine‐binding proteins, and/or heptahelical receptors capable of interfering with parts of the host chemokine system; or they contain genes encoding chemokines and/or chemokine receptors that activate, or are activated by, host cCKRs or chemokines 129. The saliva of blood‐sucking ticks contains chemokine‐binding proteins thought to suppress inflammation at the bite site 130, 131, 132. Human immunodeficiency virus (HIV) has evolved to exploit cCKRs: CXCR4, and particularly CCR5, are vital coreceptors mediating HIV entry into cells and can dock to the HIV gp120 envelope protein after it has bound CD4 133, 134, 135, 136, 137, 138, 139. The ligands for these cCKRs block HIV entry into cells by steric hindrance or cCKR down‐regulation, and genetic variation in genes encoding CXCL12, CCR5, and CCR5 ligands profoundly influences susceptibility to HIV infection and the rate of progression to AIDS 3, 7, 140. Most notably, homozygosity for the nonfunctional Δ32‐*CCR5* allele profoundly protects against HIV infection, while Δ32‐*CCR5* heterozygosity is associated with slowed progression to AIDS in most cohorts of HIV‐infected people 7, 140, 141, 142. CCR5 antagonist Maraviroc 143 is now used clinically, alongside other drugs, to delay progression to AIDS in HIV‐positive patients, and there was considerable publicity when transplantation of HLA‐matched stem cells from Δ32‐*CCR5* homozygotes proved very effective in treating an HIV‐infected patient 144. However, some caution is required because after infection with West Nile Virus, Δ32‐*CCR5* homozygosity increases the likelihood of developing encephalitic symptoms, and of dying from the infection 145, most likely due to defects in the trafficking of protective leukocytes into the brain 146.

Other pathogens target chemokine receptors. Some malarial parasites use ACKR1 to enter erythrocytes (see below); cyclophilin‐18 from *Toxoplasma gondii* binds to, and signals through, CCR5 147; respiratory syncytial virus (RSV) uses it's glycoprotein G to infect a subset of B cells through CX_3_CR1 148; and *Staphylococcus aureus* leukotoxin ED (LukED) and γ‐hemolysin AB toxin (HlgAB) target ACKR1 and various cCKRs to lyse erythrocytes and kill leukocytes, respectively 149, 150, 151, 152. These interactions have important consequences for the pathogenicity of these microorganisms.

### Atypical chemokine receptors

Atypical chemokine receptors, of which there are four (yellow boxes, Fig. 1), are structurally related to cCKRs but do not couple to many, if any, of the signal transduction pathways activated by cCKRs 66, 153. Many publications report signaling and associated biological responses via ACKR3, but it remains unclear and/or controversial whether ACKR1, 2, and 4 can transduce signals at all after chemokine binding 154. This may be in part due to the absence, or modification, of appropriate signaling motifs on the intracellular surface of ACKRs, such as the canonical DRYLAIV motif present in the second intracellular loop of cCKRs 153. However, ACKRs bind chemokines with high affinity 154, and, like cCKRs, use sulphated tyrosine residues to enhance chemokine binding 155, 156.

All ACKRs appear to be involved in regulating chemokine localization, distribution, and abundance, thereby indirectly controlling interactions between chemokines and cCKRs 153. For example, ACKR1 transports chemokines across endothelial cells for presentation to blood‐borne leukocytes 157, 158, and, on erythrocytes, ACKR1 buffers chemokine abundance in the blood 159, 160, 161, 162, 163: this likely prevents cCKRs on circulating leukocytes being inappropriately desensitized by exposure to excess chemokine. Interestingly, ACKR1 can regulate hemopoietic stem and progenitor cells in the bone marrow (BM), and control neutrophil phenotype/abundance in the blood, although the underpinning molecular mechanisms remain unclear 164, 165. ACKR1 is pirated by malarial parasites *Plasmodium vivax* and *Plasmodium knowlesi*, which use their Duffy binding protein (DBP) to engage ACKR1 and gain entry into erythrocytes 166, 167, 168. Genetic variation in *ACKR1* profoundly influences susceptibility to infection by these parasites, and the complete loss of ACKR1 from erythrocytes is very common in sub‐Saharan African populations 169, 170. The absence of ACKR1 from erythrocytes also appears to cause benign ethnic neutropenia 165, 171, 172 and may influence HIV infection by leading to CCR5 ligand dysregulation or loss of ACKR1‐mediated HIV presentation 173, 174, although this was not borne out in other studies 175, 176, 177, 178.

ACKR2 is a well‐characterized chemokine scavenger. It constitutively shuttles to and from cell surfaces without needing chemokine‐induced signals to do so, and internalizes any chemokines it encounters while exposed to the extracellular space 179, 180. Internalized chemokine is dislodged from ACKR2 and degraded 179, 180. ACKR2 serves key regulatory functions in developing mammary gland and on lymphatic endothelial cells (LECs), innate‐like B cells, and placental trophoblasts 181, 182, 183, 184, 185, 186, 187, 188, 189, 190. ACKR3 is instrumental in controlling the CXCL12‐CXCR4 axis, either by scavenging CXCL12 or by heterodimerizing with, and regulating the function of, CXCR4 99, 100, 191, 192, 193, 194, 195, 196, 197, 198, 199, 200. ACKR4‐mediated scavenging of CCL19 and/or CCL21 can regulate CCR7‐dependent dendritic cell migration and adaptive immune responses 201, 202, 203, 204, and roles for this ACKR have been reported in the thymus 205, 206. cCKRs can also remove extracellular chemokines, albeit less efficiently than ACKRs, and internalization of surface chemokine/cCKR complexes is a key aspect of cCKR regulation. Consequently, mice in which a cCKR gene has been deleted can have elevated levels of the chemokine(s) that normally bind that cCKR 207. Thus, chemokine regulation by receptor‐mediated internalization is not limited to ACKRs.

## The function of the chemokine network

**Figure 2:**
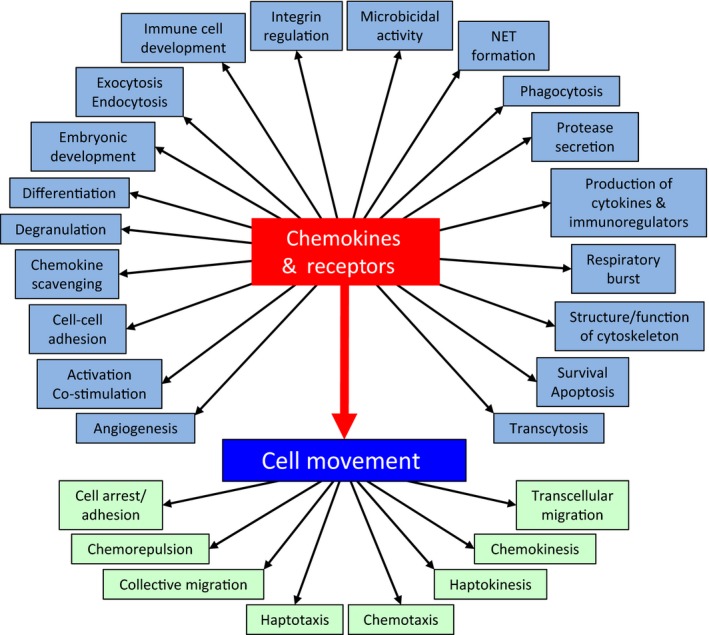
Functions of chemokines and their receptors. Biological processes reported to be regulated by chemokines and their receptors are in light blue boxes arranged clockwise, in alphabetical order (starting bottom left), around the central ‘Chemokines & Receptors’ box. ‘Cell Movement’ is depicted as the dominant biological process regulated by chemokines and their receptors: chemokine‐mediated cell arrest/adhesion, and the different types of migratory behavior known to fall under chemokine control, are shown in light green boxes.

By far the most studied function of the chemokine network is cell migration, particularly of leukocytes. However, the biological activity of chemokines is by no means limited to this function or to these cell types (Fig. 2). As recently reviewed elsewhere 208, a wide variety of other biological processes can be induced by the activation of cCKRs on leukocytes, including proliferation, survival, differentiation, cytokine production, degranulation, and respiratory burst (Fig. 2). Moreover, several chemokines have direct antimicrobial activity 209. In addition, many nonleukocytic cell types, including neurons, astrocytes, epithelial cells, mesenchymal cells, and endothelial cells, can express cCKRs and respond in a wide variety of ways to chemokines 210, 211, 212, 213, 214, 215, 216. For example, many chemokines directly regulate angiogenesis, with distinct subsets showing negative or positive angiogenic activity 214, 215. Interestingly, cancer cells of nonleukocytic origin can evolve to express cCKRs and respond to chemokines: this can encourage local invasion, spread to draining lymph nodes, and the metastatic seeding of distant tissues 217, 218. Cell movement is the dominant biological process regulated by chemokines and their receptors, and many different types of cell movement that have been reported fall under chemokine control, including chemotaxis (often seen as the classic chemokine‐driven form of migration) and also encompassing haptotaxis, chemokinesis, haptokinesis and transcellular migration. In some contexts, chemokines can direct the migration of groups of cells (referred to as collective migration) 219, 220, 221, or stimulate cell adhesion, causing cell movement to stop. There are also reports of cells moving down, rather than up, chemokine concentration gradients, i.e., away from the chemokine source, a process termed chemorepulsion or chemofugetaxis 222, 223, 224 (Fig. 2).

Leukocyte migration is of critical immunological importance. Leukocytes must be in the right place at the right time so that their immunological functions can be appropriately localized and directed. Immune surveillance requires the continuous trafficking of leukocytes out of BM and into, within, and out of the other tissues of the body. When tissues become damaged and/or infected, the rapid recruitment of innate immune cells is essential to kill pathogens, prevent microbial dissemination, drive inflammation, and help repair damage. The elaboration of a regulated adaptive immune response, and the subsequent development of immune memory, depends on further carefully choreographed leukocyte migratory processes. Chemokines are of central importance in all these processes driving leukocytes into and out of blood and lymphatic vessels, and directing their interstitial movement and positioning. Without chemokine‐directed leukocyte migration, immune tolerance breaks down, immunosurveillance fails, and protective immune responses are compromised. However, chemokine‐directed leukocyte migration also contributes to diseases that have an immune or inflammatory component including autoimmunity, allergy, chronic inflammatory disease, atherosclerosis, cancer, and many others. In this context, interfering with chemokine‐directed leukocyte migration has therapeutic potential.

**Figure 3:**
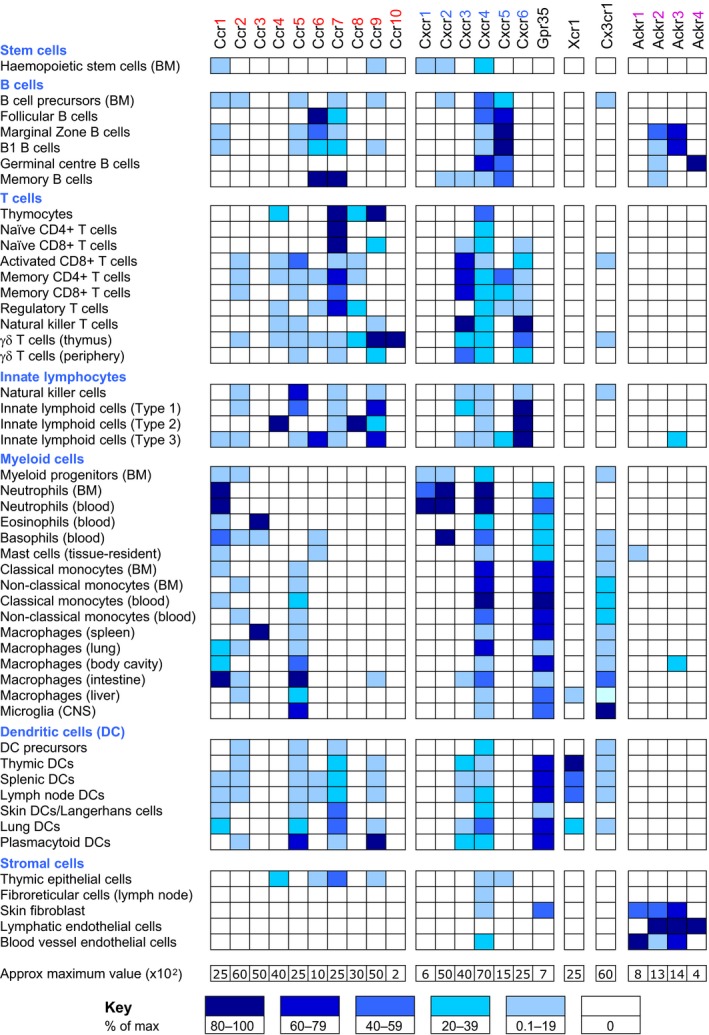
Expression of chemokine receptor genes in selected mouse leukocytes and stromal cells. The figure was generated using transcriptomic data from The Immunological Genome Project database (http://www.immgen.org) 351. The maximum expression value was identified for the cell types shown and is indicated in the row at the bottom of the Figure in arbitrary units. For each receptor, this value was set to 100%. Estimated background values (typically between 50 and 100) were determined by examining expression graphs for all cell types on the database. Expression in the cell types shown in the left hand column was then assigned a color according to the percentage of the maximum expression value (see Key on right). Note that not all cells in a cell population will necessarily express the receptor. BM, bone marrow; DC, dendritic cell.

The size of the chemokine and cCKR families enables leukocyte recruitment to be tailored to fit the immunological needs of tissues. While many molecules are required for cells to be able to leave the bloodstream and navigate within tissues 29, 225, typically the expression of a particular cCKR enables a leukocyte to migrate in response to that receptor's ligands. Figure 3 shows the expression of chemokine receptor genes in a variety of leukocytes and stromal cells in mice. These data are consistent with protein expression data, and broadly conserved in humans. Some cCKR genes, such as *Ccr3*,* Cxcr1*, and *Cxcr2*, clearly show highly restricted patterns of expression, while others, particularly *Cxcr4*, are more uniformly expressed. ACKRs are mainly limited to stromal cells, although *Ackr1* is expressed by erythrocyte precursors (not shown in Fig. 3) 164 and the other ACKRs are transcribed in discrete subsets of B cells 99, 189, 226.

**Figure 4:**
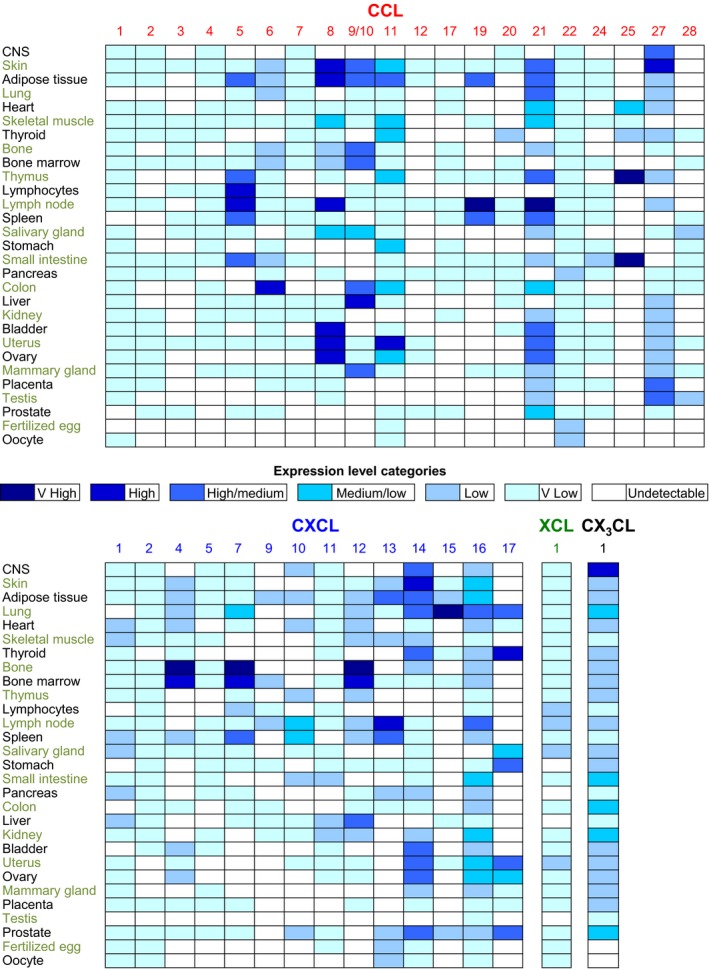
Expression of chemokine genes in selected mouse tissues under steady‐state conditions. By examining graphs generated on The Immunological Genome Project website (http://www.immgen.org) 351 using their transcriptomic data, the expression of each chemokine, in each of the tissues shown (left hand column), was assigned to one of the seven color‐coded ‘Expression Level Categories’ indicated in the key in the center of the Figure. Expression in the central nervous system (CNS) was estimated by examining ImmGen data on numerous component parts of the CNS. Expression by lymphocytes was included to help indicate whether chemokine expression by secondary lymphoid organs could be attributed to expression by lymphocytes, the dominant cell type in these organs. It was estimated by examining ImmGen data on B cells, CD4+ T cells, and CD8+ T cells. It should be noted that the function of a chemokine in a tissue will depend of where it is expressed: for example, expression by blood vessel endothelial cells may enable leukocyte recruitment from the blood, while their presence elsewhere might help direct leukocytes to specific microanatomical niches, or encourage departure via lymphatic vessels.

Leukocyte activation can change cCKR expression profiles dramatically to couple changes in immunological function with switches in migratory potential. This makes sense if the new function requires the cell to localize to a different tissue or microanatomical niche. This is discussed below in the context of CD4+ T cells. Likewise, distinct tissues or tissue domains express specific profiles of chemokines under homeostatic conditions (Fig. 4), and this changes with infection or damage when the immunological requirements of the tissue change. For example, in mice, CCL25 is constitutively expressed in the small intestine 227, while mouse skin makes substantial quantities of CXCL14, CCL8 ,and CCL27 10, 228, 229, but when these or other tissues are damaged, inflamed or infected, large numbers of inflammatory chemokines are induced to direct the rapid recruitment of innate immune cells and the subsequent homing of effector T cells.

## Development, homeostasis, and immune surveillance

Only the CXCL12/CXCR4/ACKR3 node of the chemokine network is necessary for life. CXCL12, the most primitive chemokine, has been strongly conserved through evolution. Acting through CXCR4 and regulated by ACKR3, CXCL12 is critical for the development of the heart, brain, vascular system, hematopoietic system, germ cells, and, in fish, the lateral line [99, 191, 192, 193, 194, 195, 196, 197, 198, 199, 200, 230, 231, 232]. Deletion of *Cxcl12* or *Cxcr4* in mice results in a variety of developmental abnormalities and death *in utero* 230, 231, 232. *Ackr3* deficiency has a similar outcome, although some *Ackr3*‐deficient mice survive until birth 99, 193, 194. ACKR3‐mediated scavenging of adrenomedullin (ADM) may be significant during heart and lymphatic vasculature development 233. Many indispensable functions for CXCL12 have been defined in the adult, including its role in hematopoiesis where it is a key component of the niche that supports hematopoietic stem cells (HSCs) in the BM 234, 235, 236. Blocking CXCR4 function liberates HSCs from the BM, and CXCR4 antagonist AMD3100 (Plerixafor) is used clinically to mobilize HSCs for collection from peripheral blood prior to autologous stem cell transplantation 237.

Autosomal dominant mutations in CXCR4 are responsible for WHIM syndrome (warts, hypogammaglobulinemia, immunodeficiency, myelokathexis syndrome), a rare genetic disease in which patients have IgG antibody deficiency, neutropenia (due to retention of neutrophils in the BM), and increased susceptibility to bacterial and viral infections (including human papillomaviruses, which causes warts) 238. The mutations truncate or mutate the C terminus of CXCR4 238, 239, disrupting negative regulatory domains and enhancing receptor activity 240, 241. Plerixafor shows promise as a therapeutic 242 and there is a remarkable report that chromothripsis, a process in which chromosomes undergo extensive rearrangements and deletions, spontaneously cured a WHIM syndrome patient 243.

CXCR5 and CCR7 serve key developmental roles by regulating the homing of lymphoid tissue inducer (LTi) cells. During embryonic life, these cells migrate out of the blood into sites where secondary lymphoid tissues will form. This is critical for the development of lymph nodes and Peyer's patches, and mice defective in both the CXCR5/CXCL13 and CCR7/CCL21 axes lack Peyer's patches and virtually all lymph nodes 244, 245, 246, 247, 248, 249.

Some cCKRs serve well‐defined homeostatic tissue‐specific functions driven by the constitutive expression of their ligands under steady‐state conditions (Fig. 4). For example, several chemokine receptors, including CCR4, CCR9, and particularly CCR7, contribute to T‐cell development by enabling cells to enter and navigate within the thymus 250, 251, 252, 253, 254, 255, 256, 257, 258, 259, 260. This facilitates the selection and differentiation processes that are essential for central tolerance, the generation of the naïve T‐cell repertoire, and natural regulatory T‐cell (nTreg) formation. Deletion of *Ccr7* or its ligands disrupts thymocyte trafficking in mice creating an aberrant naïve T‐cell repertoire that drives autoimmunity 252, 253, 254, 255, 261. CCR7 is also essential for leukocyte entry into lymph nodes and other secondary lymphoid tissues 262, with recent reports describing circadian fluctuations in the CCL21/CCR7 axis and lymphocyte trafficking into lymph nodes controlled by adrenergic nerves 263, 264, 265. CCR7 facilitates lymphocyte recruitment from blood 266; stimulates intranodal T‐cell motility 267, 268, 269 and retention 264, 270; directs dendritic cells (DCs) 27 and other leukocytes 271, 272 into tissue lymphatic vessels along CCL21 gradients 27 aided by DC‐induced CCL21 secretion from LECs 273; mediates intralymphatic crawling 274, 275; and allows DCs to enter the lymph node parenchyma from the subcapsular sinus 276. Thus, in addition to directing central tolerance, CCR7 is essential for peripheral tolerance and the initiation of adaptive immune responses 262.

There are many other instances of chemokine‐driven homeostatic leukocyte trafficking. For example, CCR2 is required for Ly6C^hi^ monocyte release from BM 277; CCR3 controls steady‐state eosinophil distribution 278; CXCR2 and CXCR4 direct neutrophil egress from, and return to, the BM 279, 280; monocytes use CX_3_CR1 to patrol blood vessel walls 281; and CCR9 regulates plasmacytoid DC and intraepithelial γδ T‐cell abundance in the small intestine 258, 282. B cells are specifically directed to lymphoid tissue follicles by CXCR5 244, 245, which also controls marginal zone B cell and B1 B cell migration in mouse spleen and body cavities, respectively 283, 284.

Therefore, chemokine‐driven cell migration serves critical developmental functions; ensures immunological tolerance is established and maintained; enables antigen‐specific lymphocytes to enter and survey antigen‐presenting cells in lymphoid tissue; and distributes leukocytes around the body so they are appropriately placed to respond to immunological challenge.

## Infection, inflammation, and immunopathology

Chemokines, along with an array of other proteins, peptides, lipids, and microbial products, direct leukocyte recruitment into infected or damaged tissues 66, 285. Many chemokines are highly inducible and produced in large quantities in response to a broad array of infectious and inflammatory stimuli. Leukocytes recruited by chemokines early to damaged or infected tissues can produce other chemokines that contribute to the next wave of leukocyte homing 286, 287. Chemokines are produced by many diseased tissues, including those affected by autoimmunity 288, 289, 290, allergy 291, Alzheimer's disease 292, chronic inflammatory disease 293, cardiovascular disease 294, and cancer 295. These inflammatory chemokine profiles typically include those chemokines that bind to the promiscuous cCKRs and ACKRs (i.e., CCR1, CCR2, CCR3, CCR5, CXCR1, CXCR2, CXCR3, ACKR1, and ACKR2) (Fig. 1), but other chemokines can also be induced, and the precise chemokine profile in a given tissue will depend on the exact nature of the inducing stimuli, the phase of the response, and the genetics of the chemokine network in the affected individual.

Inflammatory chemokine abundance, distribution, and activity will be controlled by their interactions with ECM, proteases, and other proteins within the tissue, and by scavenging via cCKRs and ACKRs, particularly ACKR1 and ACKR2. Inflammatory chemokines make a major contribution to the recruitment of leukocyte populations required to meet the immunological needs of affected tissues, and will regulate any nonleukocyte tissue cells (epithelial, mesenchymal, and/or endothelial) constitutively or inducibly expressing cCKRs. Many microbes have evolved to interfere with inflammatory chemokines and cCKRs, leading to an ‘arms race’ that has built robustness and redundancy into this part of the chemokine network. Nonetheless, individual cCKRs serve indispensable roles in a variety of contexts, and there is a vast literature describing how deleting, inhibiting, or blocking individual cCKRs or ACKRs impacts on a wide variety of experimentally induced immune and inflammatory responses, and modifies pathology in a diverse array of animal models of human disease. This understandably led to the development of small molecule cCKR antagonists to trial in patients with immune or inflammatory disease 66. However, despite considerable efforts, to our knowledge, no effective therapeutics for these diseases have yet emerged, and while the in‐built robustness of the inflammatory chemokine network has no doubt been a contributing factor, several other likely reasons have been proposed and discussed 296. Nonetheless, clinical trials using chemokine receptor antagonists, or other therapeutic approaches that target or exploit chemokines, continue to take place.

## Chemokine receptor switching

Changes in leukocyte function are intimately associated with switches in cCKR expression. For example, DC trafficking from tissues to draining lymph nodes requires inflammatory cCKRs to be lost and CCR7 to be switched on 262, 297, 298, 299. cCKR switching is also prominent during CD4+ and CD8+ T‐cell activation and differentiation, and cCKRs are reported to directly contribute to T‐cell costimulation 300, 301. When CD4+ T cells encounter antigen, they can differentiate into one of many functionally distinct T cell types, including effector T cells (Th1, Th2, and Th17), follicular T cells (Tfh and Tfr), induced regulatory T cells (iTreg), and memory T cells (Tcm and Tem). These cells have discrete immunological functions that require specific migratory behaviors so they need to express particular cCKR profiles. Th1 cells typically express CCR5 and CXCR3 (as do many recently‐activated CD8+ T cells); Th2 cells preferentially display CCR3 and CCR4; and Th17 are often CCR6+ 302, 303, 304, 305, 306, 307, 308. This enables these effector T cells to home to infected or inflamed tissues where they contribute to microbial clearance and tissue repair. In contrast, Tfh and Tfr cells control activated B cells so need to enter B cell follicles in lymphoid tissues: they achieve this by up‐regulating CXCR5 expression 309, 310, 311, 312, 313, 314. Likewise, during viral infection, CXCR5 is expressed by some activated cytotoxic T cells so that they enter follicles to attack virally infected Tfh and B cells 315.

Antigen‐experienced T cells can be imprinted with cCKRs that enable them to selectively home to specific tissues. T cells that encounter antigen in mesenteric lymph nodes draining the small intestine will often express CCR9 to enable homing back to the small intestine 316, 317, 318, 319. This depends on the specialization of DC and stromal cells in the mesenteric lymph nodes and production of retinoic acid from dietary vitamin A 319, 320, 321, 322, 323, 324. Likewise, T cells activated in skin‐draining lymph nodes typically express CCR4, CCR8, or CCR10 to enable homing to the skin: this may depend on skin‐derived vitamin D_3_ and keratinocyte products 10, 229, 325, 326, 327, 328, 329. In contrast, central memory CD4+ and CD8+ T cells (Tcm), like naïve T cells, traffic through secondary lymphoid organs using CCR7, while other memory T cells lose CCR7 and express cCKRs that enable them to home to nonlymphoid tissues 330. These memory cells can remain resident in the nonlymphoid tissue or home back to lymph nodes by up‐regulating CCR7 271, 272. In addition, recent work has defined three functionally distinct subsets of antigen‐experienced CD8+ T cells based on their differential expression of CX_3_CR1 331.

Antigen‐experienced B cells also use chemokine receptor switching to direct differentiated cells to discrete tissues or tissue domains. CCR7 up‐regulation directs activated B cells to the boundary of the follicle and the T‐cell area 332. CXCR4 and CXCR5 are involved in the movement of antigen‐experienced B cells in germinal centers, and CCR6 expression marks memory B‐cell precursors in these structures 333, 334, 335. CXCR4 homes long‐lived plasma cells to supportive niches in BM and spleen 336, 337, and CCR9 and CCR10 direct plasma cell homing to the intestine and mammary gland 338, 339, 340, 341, 342, 343.

## Concluding remarks

The chemokine network is enormously complex, comprising of a large number of interacting ligands, receptors, and regulatory proteins engaged in overlapping and diverse cellular processes. The induction of migration, particularly of leukocytes, is its central biological purpose, but its influence extends far beyond this. The physiological contribution of the chemokine network is substantial, with fundamental roles in development, homeostasis, immune surveillance, inflammation, protection from infection, tissue repair, and innate and adaptive immunity. Virtually all diseases involve chemokines and their receptors in some way, some more prominently than others, and although clinical translation has been slow, drugs targeting cCKRs have successfully made it to clinic. The first chemokine was discovered over 40 years ago, and our understanding of the chemokines and their receptors is now well advanced. Nonetheless, there is still much to uncover, and chemokines and their receptors are likely to remain prominent in the scientific literature for years to come.

## Author contributions

Both authors planned the article structure and content. RJBN wrote the text and constructed the Figures. CH reviewed and edited the article, and helped with literature search and referencing.
